# Curcumin Stimulates the Overexpression of Virulence Factors in *Salmonella enterica* Serovar Typhimurium: In Vitro and Animal Model Studies

**DOI:** 10.3390/antibiotics11091230

**Published:** 2022-09-10

**Authors:** Martin Zermeño-Ruiz, Itzia A. Rangel-Castañeda, Daniel Osmar Suárez-Rico, Leonardo Hernández-Hernández, Rafael Cortés-Zárate, José M. Hernández-Hernández, Gabriela Camargo-Hernández, Araceli Castillo-Romero

**Affiliations:** 1 Departamento de Microbiología y Patología, Centro Universitario de Ciencias de la Salud, Universidad de Guadalajara, Calle Sierra Mojada 950, Independencia Oriente, Guadalajara 44100, Mexico; 2 Centro de Investigación y Estudios Avanzados del Instituto Politécnico Nacional, Ciudad de México 07360, Mexico; 3 Departamento de Fisiología, Centro Universitario de Ciencias de la Salud, Universidad de Guadalajara, Calle Sierra Mojada 950, Independencia Oriente, Guadalajara 44100, Mexico; 4 Departamento de Ciencias de la Salud, Centro Universitario de los Altos, Universidad de Guadalajara, Av. Rafael Casillas Aceves No. 1200, Tepatitlán de Morelos 44100, Mexico

**Keywords:** *Salmonella enterica* serovar Typhimurium, curcumin, antibacterial activity, pathogenicity, *C. elegans*

## Abstract

*Salmonella* spp. is one of the most common food poisoning pathogens and the main cause of diarrheal diseases in humans in developing countries. The increased *Salmonella* resistance to antimicrobials has led to the search for new alternatives, including natural compounds such as curcumin, which has already demonstrated a bactericidal effect; however, in Gram-negatives, there is much controversy about this effect, as it is highly variable. In this study, we aimed to verify the antibacterial activity of curcumin against the *Salmonella enterica* serovar Typhimurium growth rate, virulence, and pathogenicity. The strain was exposed to 110, 220 or 330 µg/mL curcumin, and by complementary methods (spectrophotometric, pour plate and MTT assays), we determined its antibacterial activity. To elucidate whether curcumin regulates the expression of virulence genes, *Salmonella* *invA*, *fliC* and *siiE* genes were investigated by quantitative real-time reverse transcription (qRT-PCR). Furthermore, to explore the effect of curcumin on the pathogenesis process in vivo, a *Caenorhabditis elegans* infection model was employed. No antibacterial activity was observed, even at higher concentrations of curcumin. All concentrations of curcumin caused overgrowth (35–69%) and increased the pathogenicity of the bacterial strain through the overexpression of virulence factors. The latter coincided with a significant reduction in both the lifespan and survival time of *C. elegans* when fed with curcumin-treated bacteria. Our data provide relevant information that may support the selective antibacterial effects of curcumin to reconsider the indiscriminate use of this phytochemical, especially in outbreaks of pathogenic Gram-negative bacteria.

## 1. Introduction

Diarrheal disease is an important global health problem and is the third cause of child mortality [1]. *Salmonella* is one of the most frequent bacteria causing diarrheal diseases [2]. *Salmonella enterica* serotypes include numerous pathogens of warm-blooded animals, including humans. *Salmonella enterica* serovar Typhimurium (*S.* Typhimurium) has been considered the prototypical broad-host-range serotype. It is a frequent cause of acute self-limiting food-borne diarrhea in numerous species, including humans, livestock, domestic fowl, rodents, and birds [3]. Recently, nontyphoidal *Salmonella* variants were associated with invasive systemic disease and high mortality rates within immunocompromised patients. *S.* Typhimurium and Enteritidis are the most usual causes of invasive disease in sub-Saharan Africa [4,5]. The pathogenicity of *Salmonella enterica* infections is expressed in three ways, such as host cell invasion, intracellular survival, and colonization. All these processes are regulated by the virulence genes located in *Salmonella* pathogenicity islands (SPI). The most extensive invasion mechanism requires the type III secretion system encoded in the SPI-1. This system is composed of a needle-like structure that injects bacterial effector proteins into epithelial cells, such as the invasion protein A (InvA). InvA is widely studied as a virulence factor; it is required to cross the epithelial cells, thus initiating infection [6,7]. Another entry mechanism involved bacterial motility. Recent data demonstrated that the deletion of flagellin gene *fliC*, which encodes the major component of the flagellum in *S.* Typhimurium, affects the entry of *Salmonella* into the host cell [8,9]. In addition, novel members of the non-fimbrial adhesins encoded in SPI-4 have been found. In a murine model, SPI-4 contributed to intestinal inflammation, via the secretion of SiiE that mediates the *Salmonella* adhesion to the epithelial cell’s surface [10]. 

Specific antimicrobial therapy ameliorates the course of illness with these pathogens. However, because of the problem of antibiotic resistance, alternative approaches have been directed toward therapies based on traditional plant medicines. 

Due to its multi-faceted pharmacology, many studies have evaluated the possible use of curcumin (CUR) to treat or prevent bacterial infections. Several studies showed this phytochemical, alone or combined with some nanomaterials or compounds, demonstrated different responses of curcumin on Gram-positive and Gram-negative bacteria. In Gram-negative bacteria, curcumin exhibits extremely low antibacterial activity [11,12,13,14,15,16,17,18,19,20,21,22]. In addition, Marathe and coworkers (2010) proved in a murine model that CUR enhances the pathogenicity of *S*. Typhimurium via regulating their defense pathways [23]. While there appear to be countless therapeutic benefits to curcumin, its effects on Gram-negative bacteria are still poorly understood and controversial.

On the other hand, the *Caenorhabditis elegans* genome can encode several antimicrobial proteins, such as caenopores, lysozymes, lectins and ABF peptides (antibacterial factors), that have a broad antimicrobial spectrum for Gram-positive bacteria [24]. Additionally, the finding that diverse bacteria are pathogenic to *C. elegans* opens the prospect of using this experimentally simple model to study microbial pathogenesis [25]. In this work, we described the role of CUR in the pathogenicity of *S.* Typhimurium. Our results demonstrated that CUR increases cell proliferation and induces *S.* Typhimurium virulence factors overexpression. Consequently, the lifespan of *C. elegans* was reduced.

## 2. Results

### 2.1. PCR Identification of Genes Encoding Specific Virulence Factors of S. Typhimurium

**Figure 1:**
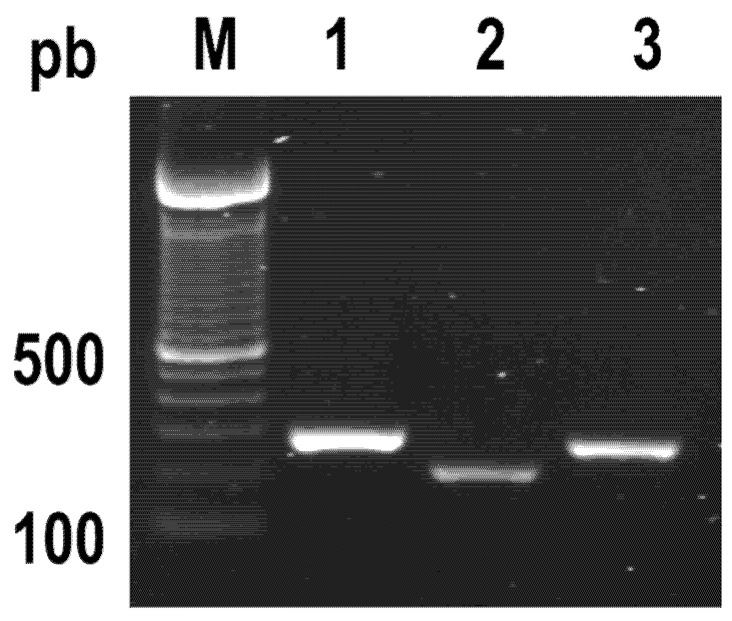
PCR detection of *Salmonella* Typhimurium virulence genes. Lane 1, *invA* gene (1322 bp). Lane 2, *siiE* gene (240 bp), and Lane 3 *fliC* gene (307 bp). M, DNA ladder, standard molecular size marker.

Virulence factors are essential for the ability of bacteria to cause disease. *Salmonella* has the ability to survive long-term frozen storage; however, it has been reported that isolated virulent bacterial strains became avirulent during storage or passages. The above is probably due to the loss of virulence plasmids [26]. A polymerase chain reaction (PCR) method confirmed that our storage and growth conditions did not affect the presence of virulence factors from the bacterial strain. Figure 1 shows the presence of three genes of *S.* Typhimurium involved in epithelial cell adhesion and invasion (*invA*, *fliC* and *siiE*).

### 2.2. Curcumin Did Not Show an Antibacterial Effect

**Figure 2:**
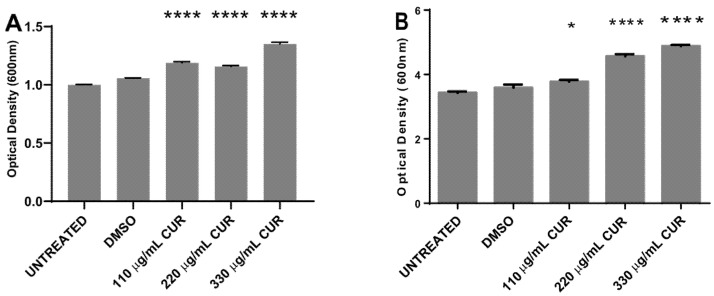
Dose–time response curves comparing curcumin (CUR) treatment in *S. enterica* ser. Typhimurium growth (OD600), and for cells incubated in the presence of dimethyl sulfoxide (DMSO). Curves are representative of at least 3 different assays. (**A**) After 4 h of incubation. (**B**) After 12 h of incubation. (* *p* < 0.05, **** *p* < 0.0001).

**Figure 3:**
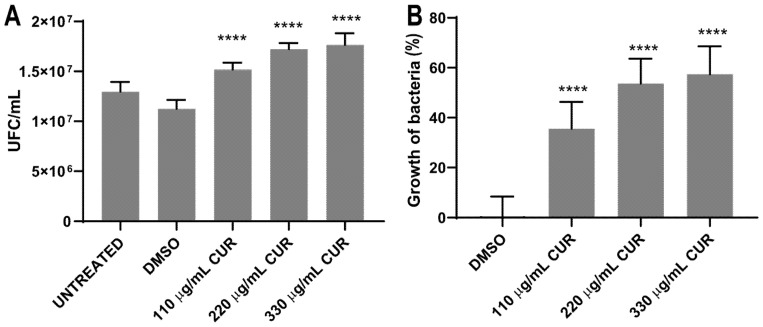
Number of colonies on agar plates (**A**) and percentage growth of *S. enterica* ser. Typhimurium (**B**), after treatment with CUR. The average and standard deviation values of six replicates are shown for the strain. (**** *p* < 0.0001).

**Figure 4:**
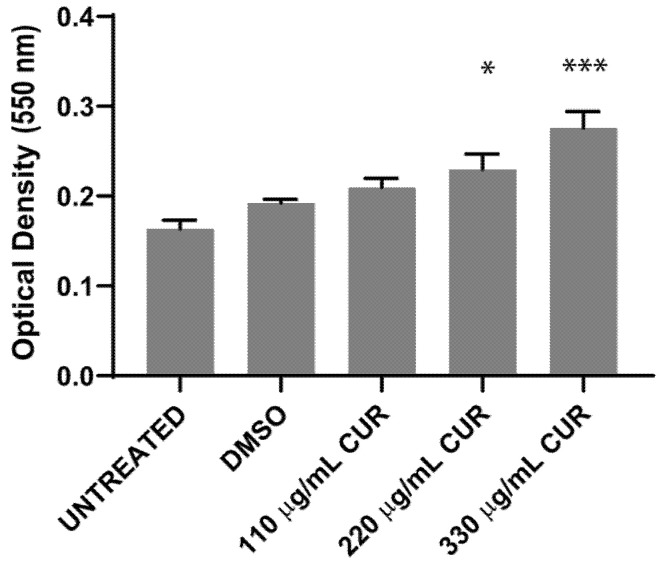
MTT reduction by *S. enterica* ser. Typhimurium treated with CUR, (* *p* < 0.05, *** *p* < 0.001).

To determine whether CUR kills *S.* Typhimurium and to investigate the effect of different environmental or growth conditions on bacteria cell survival, 10^7^ CFU/mL was exposed to dimethyl sulfoxide (DMSO) or CUR for 2 h, and the effect was evaluated by spectrophotometric (OD600), pour plate and MTT assay. The results, presented in Figure 2, indicate that the treatment with 110, 220 and 330 μg/mL of CUR did not inhibit bacterial growth. After 4 h of incubation, curcumin provoked a significant dose-dependent growth stimulation. Untreated and DMSO-treated Salmonella strains reached the exponential growth phase with similar growth rate (Figure 2A). After 12 h of incubation, *Salmonella* maintained a significant growth increase in the presence of CUR (DMSO 3.6 ± 0.09 SD vs 110 μg/mL 3.8 ± 0.05 SD, 220 μg/mL 4.6 ± 0.06 SD and 330 μg/mL 4.9 ± 0.04 SD) (Figure 2B). By the pour plate, there was an increment in the number of colonies with CUR following a dose-response profile (Table 1, Figure 3A). The percentage of overgrowth in *S.* Typhimurium increased significantly from 35% to 57% (Figure 3B). These results are consistent with the MTT assay, where formazan cell viability/crystal formation increases with curcumin concentrations. When formazan was solubilized, the absorbance at 550 nm of curcumin-treated cultures was higher than the negative controls, maintaining the dose-dependent profile (DSMO 0.1925 ± 0.004 vs 110 μg/mL 0.2095 ± 0.01, 220 μg/mL 0.2297 ± 0.04, 330 μg/mL 0.2758 ± 0.018) (Figure 4).

### 2.3. Virulence Factors Are Upregulated by Curcumin

**Figure 5:**
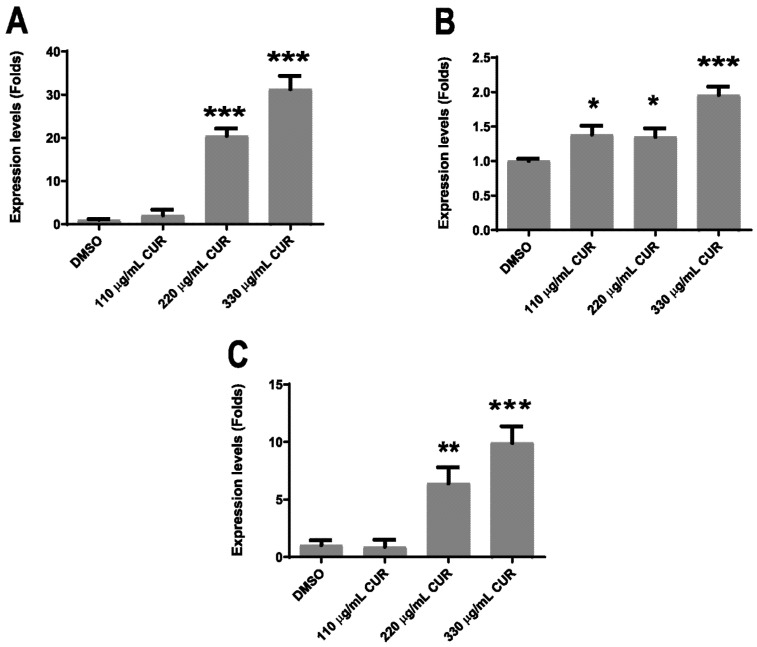
Relative–quantitative RT-PCR assay for *fliC*, *siiE* and *invA* after CUR treatment; *S. enterica* ser. Typhimurium *siiE* (**A**), *invA* (**B**) and *fliC* (**C**), mRNA expression levels (* *p* < 0.05, ** *p* < 0.01, *** *p* < 0.001).

It has been demonstrated that CUR attenuates the virulence pathogens by the downregulation of transcription of virulence genes [27,28]. We measured *fliC*, *siiE* and *invA* expression levels by relative–quantitative RT-PCR to determine whether CUR directly affected bacterial virulence. All genes showed significant gene expression changes in response to CUR treatment. In *S.* Typhimurium, treatment with CUR provoked the increase in mRNA expression for *siiE*, *invA* and *fliC*. Among the three genes, *siiE* had the higher range of mRNA expression (18- and 28-fold), with the concentrations of 220 and 330 μg/mL of CUR, respectively (Figure 5A), while *invA* had the smallest range (0.4- and 0.9-fold), with the same doses (Figure 5B). Finally, *fliC* had also significantly increased mRNA expression (5- and 10-fold) with 220 and 330 μg/mL of CUR, respectively (Figure 5C).

### 2.4. Curcumin Enhanced the Pathogenicity of S. Typhimurium in C. elegans

**Figure 6:**
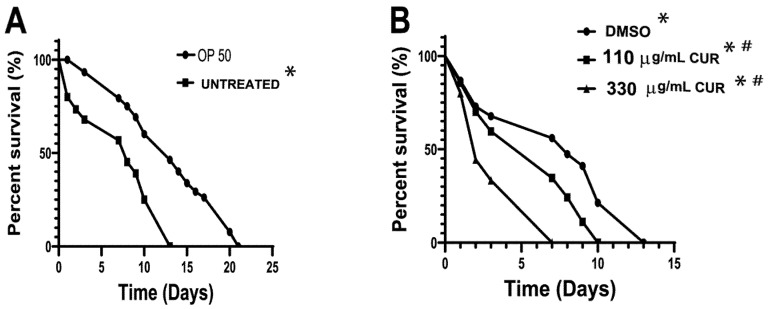
Lifespan of C. elegans infected with *S. enterica* ser. Typhimurium strain treated with 110 and 330 μg/mL of CUR (**A**). Survival curves for nematodes fed with *S*. Typhimurium and *Escherichia coli* OP50. (**B**) Life expectancy of nematodes fed with *S*. Typhimurium pretreated with DMSO, 110 and 330 μg/mL. Kaplan–Meier survival curves for *S. enterica* ser. Typhimurium. Survival percent are based on data from pathogenicity assay. Data were analyzed by log rank test, and all pairwise multiple comparison procedures used the Holm–Sidak method. n = 90, * *p* < 0.001 in relation to the OP50 and # *p* < 0.001 regarding the Untreated.

Earlier reports have shown that *S.* Typhimurium can kill *C. elegans* [24,29]. To validate nematode survival in the presence of this pathogenic strain, 8 × 10^8^ cells/mL were used as bacterial food for *C. elegans*, and nematode survival was evaluated. As a negative control, nematodes fed with *E. coli* OP50 were used. The results obtained show that the mean and maximum life expectancy of nematodes fed with the pathogenic strain decreased significantly, with respect to worms fed with the OP50 strain (negative control) (Figure 6A). To validate whether CUR increased the virulence of *Salmonella*, 8 × 10^8^ cells/mL were exposed for 2 h to DMSO and 110 and 330 μg/mL of CUR, and subsequently used as nematode food. The results show that feeding *C. elegans* with CUR-treated bacteria significantly shortens the lifespan of the nematode by 66% at the 330 μg/mL concentration compared to DMSO treatment (Figure 6B). The above effect correlates with the overexpression of virulence factors in *S.* Typhimurium due to the use of CUR. Survival curve of worms fed with DMSO-treated bacteria were not different from the curve of worms fed with untreated bacteria (Figure 6, LogRank *p* = 0.968 for *S.* Typhimurium, n = 90). OP50-fed worms showed the usual lifespan, ranging from 16 to 22 days. 

## 3. Discussion

Diarrheal diseases are one of the leading causes of death in children under 5 years and adults over 65 years. After Rotavirus, enteric bacteria are an important cause of morbidity and mortality. *S.* Typhimurium is included among the major isolated agents in developing countries [1,30]. Antibiotics are effective in life-threatening cases caused by bacterial pathogens; however, due to increased resistance and the potentially serious side effects of combinatorial therapies, there is a pressing need to have new alternatives [31,32,33,34]. In recent years, CUR, the principal and most active curcuminoid of *Curcuma longa* L. (*C. longa*), has gained considerable attention, due to its antimicrobial activity in different strains of bacteria. In 2016, Hayati Gunes et al [19] found that CUR has high antibacterial activity against *E. coli*, in relation to other bacteria, with a minimum inhibitory concentration (MIC) for CUR of 163 µg/mL. Others found that in combination with antibiotics, the CUR antibacterial activity ranges from 125 to 500 µg/mL [35]. Although most studies suggest that CUR has activity against both Gram-positive and Gram-negative bacteria [11,12,13,16,17,18,19,20,21], its activity against *S.* Typhimurium is considerably controversial. Meanwhile, in chicken, treatment with *C. longa* prevents intestinal colonization by *S.* Typhimurium [36]. In a murine model, CUR increases the pathogenicity of this bacteria [23]. In addition, reports are showing that the efficacy of antibiotics is directly related to the level of inoculum size. Bacteria might appear susceptible when the inoculum is low density (10^5^ CFU/mL) but resistant if the inoculum size is increased (high density ~10^9^ CFU/mL; depending on the clinical strains) [37,38,39]. In this report, we explore the antibacterial efficacy of CUR, and the killing assay was performed using 10^7^ CFU/mL. In the present study, even though we followed the procedure reported by Hayati Gunes et al. [19], CUR at 110, 220 and 330 µg/mL for 16–18 h at 37 °C, 250 rpm, was not active against *S.* Typhimurium (data not shown). It is well documented that the bacterial growth rate determines the bacterial susceptibility to antimicrobials; bacterial overgrowth provokes the nutrients deprivation that induces modifications of the cell envelope [40], and generally, there is no correlation with the antimicrobial concentration. Considering this, CUR treatment was performed throughout each phase of growth. Our results provide evidence that CUR did not inhibit the growth of *Salmonella*, but promoted a significant overgrowth instead, after 4 h of incubation. Many bacterial species and antibiotic classes exhibit heteroresistance, meaning that a susceptible bacterial isolate harbors a resistant subpopulation that can grow in the presence of an antibiotic. In this work, CUR was in contact with *Salmonella* for only 2 h, after which it was removed, but, interestingly, in *Salmonella*, the overgrowth continued after 12 h of incubation. This suggests that CUR enhances the speed at which cells proliferate and that the modification is transmitted to new generations. It has been reported that *S.* Typhimurium has some genes with diverged expression domains that are involved in different metabolic pathways compared with *E. coli*, leading to their better survival and propagation [41,42,43]. More studies are necessary to identify how CUR regulates the growth in *Salmonella*. On the other hand, the standard optical method for quantifying cell density (OD 600 nm) cannot distinguish live from dead bacteria or even particles. Therefore, in order to improve the results, viability and metabolic activity was validated by the pour plate method and MTT assay [44,45,46]. Our results confirm that, after 12 h of incubation, CUR does not affect the growth of *S.* Typhimurium; we have metabolic active growing cells.

CUR has been found to modulate the activity of several key transcription factors and, in turn, the cellular expression profiles [47]. In bacteria and parasites, CUR is reported to modulate the virulence factor expression [27,28]. The pathogenicity of bacteria is related to many and strain-specific virulence factors. In *Salmonella*, we analyzed three virulence genes, *invA* for the *Salmonella* genus, *fliC*, and *siiE* for Typhimurium serovar. Our result showed that all genes were found to be upregulated by CUR. For *invA*, a gene that mediates invasiveness, the overexpression was only 0.9-fold. The higher overexpression levels were observed with *fliC* and *siiE* (10- and 28-fold, respectively). In another *Salmonella* species, flagella could be dispensable for host cell adhesion, but for *S.* Typhimurium, the flagellum is a key virulence-associated phenotype. A functional flagellum is necessary for epithelial cell invasion and macrophage uptake; besides, it participates in proinflammatory cytokine expression. In the case of the adhesin SIIE, some studies show that the infection of host organisms by *Salmonella* involves the cooperative activity of the *Salmonella* pathogenicity island 1 (SPI1)-encoded type III secretion system (T3SS) and SIIE. Without the function of the SPI4 T1SS or SiiE, *Salmonella* is highly reduced in adhesion [7,48,49]. Our results suggest that CUR enhances the adhesion ability of *Salmonella*. Further studies are needed to elucidate the exact mechanism by which CUR upregulates the expression of the major virulence factors of *S*. Typhimurium [23,50,51]. The higher pathogenic potential, which bacterial strains exposed to CUR possess, was validated using the nematode *C. elegans*. It is known that *S.* Typhimurium is pathogenic to *C. elegans*; even though the nematode expresses numerous antimicrobial proteins, this bacterial strain proliferates and establishes a persistent infection in the intestine of the nematode [52]. In this study, the overexpression of virulence factors in *Salmonella* by CUR correlates with the short lifespans of *C. elegans* in a lifespan assay. The rate of mortality of *C. elegans* fed with untreated *S.* Typhimurium was similar to that found by other authors [25]; the life expectancy of the nematode was reduced by 66%, in comparison to the *E. coli* OP50 strain. In nematodes fed with CUR-treated *S.* Typhimurium, there was a direct correlation between the overexpression of virulence genes and the mortality rate; the complete mortality occurred after 10 and 7 days with 110 and 330 µg/mL, respectively, suggesting increased bacterial infection after exposure to CUR. In contrast, with other Gram-negative bacteria, it has been reported that CUR reduced the production of virulence factors, affecting the adherence and the formation of biofilm [28]. It is important to emphasize that further studies are necessary.

## 4. Materials and Methods

### 4.1. Bacterial Strain

Dr. Jeannette Barba León, Universidad de Guadalajara, kindly provided the *S.* Typhimurium (071M7) used in the current study [53].

### 4.2. Maintenance and Preservation of Microorganisms

The bacterial strain was grown in nutritive agar (plates) (Becton Dickinson, Maryland, USA) at 37 °C for 18–20 h. The cultures were stored at 4 °C, with streak plating onto fresh agar plates every seven days. A glycerol stock of bacteria was stored at −80 °C.

### 4.3. Extraction of Genomic DNA

Genomic DNA was obtained from *S.* Typhimurium cultures using the DNeasy^®^ Blood & Tissue kit (QIAGEN, Hilden, Germany), following the manufacturer’s instructions. The DNA was stored at −20 °C. Purity and concentration were determined by 1% agarose (Ultra-Pure—Agarose, Invitrogen, Carlsbad, CA, USA) gel electrophoresis and by spectrophotometry, respectively. Electrophoretic gels were stained with GelRed (Nucleic Acid Gel, Biotium, Landing Pkwy, CA, USA) and visualized on a trans-illuminator (UVP Benchtop 2UV, Fisher Scientific, Waltham, MA, USA). 

### 4.4. Presence of Virulence Genes

The pathogenicity of *Salmonella* spp. has been related to numerous virulence genes. The invasion protein InvA is one of the most studied virulence factors. Flic-encoded flagellin protein, and the giant, non-fimbrial adhesin protein SIIE have been also implicated in successful host infection [10,54,55]. The expression of *invA* (GenBank Accession M90846.1), *fliC* (GenBank Accession KF589316.1) and *siiE* (GenBank Accession AJ576316.1) was validated in the bacterial strain. A specific region of each gene was amplified from genomic DNA (DNeasy^®^ Blood & Tissue, IAGEN) by PCR using the following primers: *siiE* sense 5’-CGA CCT GAG TCA CCG TTG GGC GAT-3 and *siiE* antisense 5′- ATT GGG CTC GGC ACT GCC ACT-3′(240 bp), *invA* sense 5′- ATG CCG GTG AAA TTA TCG CCA CGT-3′, *invA* antisense 5′- ATG CCG GCA ATA GCG TCA CCT-3′(322 bp), fliC sense 5′-AAA GCC TCG GCT ACT GGT CTT GGT G -3′ and *fliC* antisense 5′- ATG CTG TGC CGG TAA CAC CTG CTG-3′(307 bp). The PCR conditions were 95 °C for 60 s, 37 cycles at 95 °C for 30 s, 72 °C for 60 s and 72 °C for 7 min. The resulting amplicons were visualized by electrophoresis in 1% agarose gel.

### 4.5. Preparation of Curcumin Stocks

The CUR was acquired from Sigma-Aldrich (≥65% (HPLC), St Louis, MO, USA). CUR stock was prepared using dimethyl sulfoxide (1.2% DMSO, Sigma-Aldrich) as a diluent and then diluted to a final concentration of 110, 220 and 330 µg/mL in phosphate-buffered saline (PBS) [56].

### 4.6. Determination of the Antibacterial Activity of Curcumin

#### 4.6.1. Spectrophotometric Method

The antibacterial activity of CUR was determined by a growing strain in Luria Bertani broth (LB) (Sigma-Aldrich, Missouri, USA), at 37 °C, 250 rpm. Cultures were allowed to grow until they reached OD600 0.08 (10^7^ colony forming units CFU/mL). Cells were pelleted by centrifugation at 1844× *g* for 5 min (Sigma 1-14K 12092 rotor), resuspended in 3 mL of PBS containing 110, 220 and 330 µg/mL of CUR and incubated for 2 h at 37 °C, 250 rpm. Untreated and 1.2% DMSO-treated cultures were used as negative controls. After the incubation period, cells were harvested by centrifugation, washed with PBS twice to remove the CUR, and grown in LB medium to achieve the exponential phase [18]. Bacterial growth (OD600) in LB medium was measured on a microplate reader (BioTek Synergy HT, Winooski, VT, USA). All experiments were performed in triplicate.

#### 4.6.2. Pour Plate Method

*S.* Typhimurium strain was exposed to DMSO, 110, 220 or 330 µg/mL of CUR for 2 h following the procedure described above. After CUR treatment, cells were harvested by centrifugation as mentioned above. The pellets were resuspended in 3 mL of PBS, and serial dilutions were performed. For the pour plate method, 100 μL of each dilution was added by pipette to the center of sterile disposable Petri dishes. Then, cooled but still molten agar medium was poured into each Petri dish. The plates were incubated overnight at 37 °C. The dilutions chosen produced between 30 and 300 separate countable colonies. The growth percentage was calculated as ((B2-A2)/A2 100)), where A is the number of colonies untreated, and B is the number of colonies in the presence of CUR. All experiments were performed in triplicate. 

#### 4.6.3. Assay MTT

According to previous reports, MTT assay modified by Wang et al. [44] was performed to determine the viability of *S.* Typhimurium after DMSO, 0, 110, 220 or 330 µg/mL of CUR exposition. Briefly, a bacterial strain was grown at 37 °C in LB broth until the OD600 reached 0.1, and then DMSO or CUR was added to each cell culture. After incubation for 2 h at 37 °C at 250 rpm, cultures were centrifuged at 1844× *g* for 5 min (Sigma 1-14K 12092 rotor). The resulting bacterial pellets were washed three times in PBS and resuspended in 1 mL of LB. Aliquots of the bacterial cultures (20 µL) were placed on 0.6 mL tubes, which had been preheated to 37 °C for 10 min. Then, 2 µL of MTT (5 mg/mL, Sigma-Aldrich, M5655 St Louis, MO, USA) was added to each tube. After incubation, for 20 min at 37 °C, the tubes were centrifuged at 10,000× *g* for 1 min (Sigma 1-14K 12092 rotor) in order to precipitate the bacteria and formazan crystals; 20 µL of the medium was removed, and the crystals were dissolved with 250 µL of DMSO. Finally, the coloration was read at 550 nm after 15 min in a microplate reader (BioTek Synergy HT, Winooski, VT, USA). All experiments were performed in triplicate.

#### 4.6.4. Statistical Analysis

All data were presented as mean values with standard deviations and analyzed using two-way ANOVA, followed by Dunnett’s multiple comparisons test (GraphPad Prism version 6.01 for Windows, GraphPad Software, La Jolla, CA, USA). *p*-values of ≤0.05 were considered significantly different.

### 4.7. Relative-Quantitative RT-PCR

The effect of CUR on the expression of *invA*, *fliC* and *siiE*, genes associated with the virulence of *S.* Typhimurium, was evaluated by semi-quantitative qRT-PCR using the primers described above. First, the bacteria strain was exposed to DMSO, 110, 220 or 330 µg/mL of CUR for 2 h following the procedure described above. After CUR remotion, they were grown overnight in LB medium. Total RNA was obtained from DMSO, or CUR treated bacterial cultures using a Total RNA Purification kit (NORGEN), following the manufacturer’s instructions. cDNAs were synthesized by a reverse transcriptase reaction (Verso cDNA Synthesis Kit, Thermo Scientific) using 1 μg of RNA and Oligo dt20 primer (Integrated DNA). Relative–quantitative RT-PCR was performed in a StepOneTM Real-Time PCR System (Applied Biosystems^TM^, Foster City, CA, USA) using Maxima SYBR Green qPCR Master Mix (Thermo Scientific) to evaluate the amplification reaction. The gene expression was normalized to the expression level of glyceraldehyde 3-phosphate dehydrogenase genes (GenBank accession no. DQ644683.1) using the following primers: gapdh sense 5′- GGT TTT GGC CGT ATC GGT CGC A-3′ and gapdh antisense 5′- ACC GGT AGC TTC AGC CAC TAC G-3′. Melting curves confirmed the absence of primer dimerization. The amplification conditions were as follows: hot start at 95 °C 10 min, 40 cycles of 95 °C 15 s, 60 °C 30 s and 72 °C 30 s. The comparative ∆∆Ct method calculated changes in expression [57]. Significant differences (defined as *p* < 0.05, indicated by asterisks in figures) were calculated by ANOVA tests using the GraphPad Prism version 6.01 for Windows (GraphPad Software, La Jolla, CA, USA). Error bars indicate standard deviations for experiments with more than one trial.

### 4.8. Maintenance and Preservation of C. elegans

The wild-type *C. elegans* variety Bristol N2 strain used in this study was provided by the Caenorhabditis Genetics Center (CGC, Minneapolis, MN, USA). Adult worms were used for all experiments, age-synchronized according to standard methods. Nematodes were grown and maintained monoxenically at 20 °C, on nematode growth medium (NGM) [58]. Animals were grown on Petri plates seeded with *Escherichia coli* strain OP50 as a food source [59,60].

### 4.9. Pathogenicity Assays of Salmonella Strains on the C. elegans Model

A pathogenicity assay is a lifespan assay, where *C. elegans* strains on NGM agar plates are fed with pathogenic bacteria (known here as killing plates) instead of the regular *E. coli* OP50 [29]. In this study, *S.* Typhimurium was grown overnight in LB at 37 °C, 250 rpm and then resuspended at an OD600 = 1. Then, cells were harvested by centrifugation, and the pellets were resuspended in 3 mL of PBS containing DMSO, 110 or 330 µg/mL of CUR and incubated for 2 h at 37 °C, 250 rpm. The killing plates were prepared by dropping 10 µL of bacterial suspension (8 × 10^8^ CFU/mL) onto NGM agar plates and incubated for 16 h at room temperature [61]. After that, 30 age-synchronized L4 worms were transferred to the killing plates. Since *C. elegans* starts laying eggs on day 1 of adulthood, the worms were transferred to fresh killing plates from the 2nd to 14th days to prevent a mistaken offspring score. In the remaining days of the trial, the transfers were carried out within a longer time (when food ran out) because the worms were in the non-reproductive phase. Worms that were alive, dead, or missing were determined and counted every other day along the time course of dying for the population, using a touch movement assay for death [62]. This assay consists of visually inspecting the worm for movement; if there is movement, then it scored as alive, and if there is no movement, even when its body is gently touched with a worm picker, it is scored as dead. Missing worms, those lost or burrowing into the medium or climbing the plate walls and drying up, were censored from the analysis. NGM agar plates seeded with untreated *S.* Typhimurium and *E. coli* OP50 were used as positive and negative controls. Every experiment was repeated three times. Data were analyzed using the Kaplan–Meier survival test and weighted log-rank tests [63]. Actual P-values are included in the figures; asterisks indicate significant differences. Differences were considered significant at *p* < 0.05.

## 5. Conclusions

This research provides new evidence on the antibacterial activity of CUR against one of the major enteric bacterial pathogens, *Salmonella*. We demonstrated that CUR increases the cell proliferation of *S.* Typhimurium, and we also observed a deregulation of three genes involved in the pathogenicity of *S.* Typhimurium leading to an increase in virulence in agreement with the results of in vivo assays. These results urge us to reconsider the indiscriminate use of CUR, especially in outbreaks of pathogenic Gram-negative bacteria.

## Figures and Tables

**Table 1:** Number of colonies on agar plates.

Sample	^A^ Average Number of Colonies Per Milliliter with Dilution 10^4^
	*S.* Typhimurium
Untreated	1.30 × 10^7^ CFU/mL
DMSO	1.13 × 10^7^ CFU/mL
CUR 110 μg/mL	1.52 × 10^7^ CFU/mL
CUR 220 μg/mL	1.72 × 10^7^ CFU/mL
CUR 330 μg/mL	1.77 × 10^7^ CFU/mL

^A^ six replicates.

## Data Availability

Not applicable.
